# Experimental and Numerical Investigation of Joints for a Pultruded Fiber-Reinforced Polymer Truss

**DOI:** 10.3390/polym14224810

**Published:** 2022-11-09

**Authors:** Yiwei Chen, Maojun Duan, Xingxing Zou, Yu Feng, Guofen Li

**Affiliations:** 1 College of Civil Engineering, Nanjing Forestry University, Nanjing 210037, China; 2 Shanghai Construction (Group) Co., Ltd., Shanghai 200080, China

**Keywords:** pultruded GFRP truss, bolted connection, bolted-bonded connection, integrated gusset plate, shear performance

## Abstract

Bolted connections usually govern the structural rigidity and load-carrying capacity of pultruded glass fiber-reinforced polymer (GFRP) truss structures. In this study, a novel bolted integrated gusset plate (IGP) connection is proposed to enhance the stiffness and capacity of GFRP truss structures. Nine double-lap shear tests of GFRP joints and numerical simulation were conducted to investigate the influence of variable design parameters of the bolted GFRP joints (number of bolts, width and thickness of GFRP, edge distance of bolts, and the employment of adhesive). Three full-scale GFRP truss joints were tested under static loading to study the response of a typical bolted connection, a bolted gusset plate connection, and the proposed IGP connection. The nine double-lap shear tests showed that the bolted–bonded mixed connection has 50% higher shear stiffness and 27% higher ductility compared with bolted joints, and bearing failure dominated the capacity of most specimens, which agreed well with numerical simulation results. Tests on the three full-scale GFRP truss joints showed that the bolted gusset plate can substantially reduce the number of cracks and improve the initial stiffness, but the maximum bearing capacity of the joints did not increase because the shear fracture of pultruded GFRP webs governs the capacity. The proposed IGP substantially increased the stiffness and capacity compared with the bolted connection and typical bolted gusset plate connection. The full-scale GFRP joint test is suggested to be used together with direct shear tests to study the performance of joints of the GFRP truss.

## 1. Introduction

Fiber-reinforced polymers (FRPs) have been made into various products and used in different civil engineering structures because of their advantages of light weight, high strength, and excellent corrosion resistance [1,2]. Different fiber types of FRP products result in different mechanical properties and corrosion, fatigue, creep resistances [3]. Typical FRPs can be divided into three main categories by fiber type: glass-, carbon- and basalt-fiber-reinforced polymers (GFRP, CFRP, and BFRP). Among them, GFRP is most widely used for its relatively low cost [4]. CFRP has outstanding strength, modulus and corrosion resistance, although the cost is relatively high. BFRP has good insulation, corrosion resistance, high temperature resistance, and recyclability [5]. Among various manufactory techniques (hand layup, pultrusion, resin transfer molding, filament wound, etc.) of FRP products, pultruded GFRP profiles have been increasingly used in civil engineering structures such as railway sleepers, hollow slabs, truss bridges, and space-formed buildings in recent years [6,7,8,9]. 

GFRP truss structures demonstrate light weight, fast construction, and extraordinary durability. Joints that connect different GFRP truss members play a critical role in transferring loads and ensuring structural integrity [10]. In field applications of truss bridges and buildings with pultruded GFRP profiles, joints are typically connected by bolted or bolted-bonded mixed connection [8]. Previous studies have revealed that GFRP joints usually govern the capacity and rigidity of the overall structure [11,12,13,14]. At present, the open and closed cross sectional profile clamping bolted connection is widely used in GFRP pultruded profile truss structures. Owing to the low shear strength of pultruded GFRP profiles, GFRP trusses are prone to brittle failure modes of web shear cracks along the profile axis under the action of bending moment within the joints [15,16]. Feng et al. conducted a full-scale test of GFRP truss joints, which revealed that the truss with GFRP pultruded profiles is a structural system suitable for bridges because of its high stiffness, bearing capacity and load efficiency, but the joints need to be further studied [8]. Keller et al. conducted experimental research on the two-span Pontresina truss bridge and showed that the span with a bolted–bonded mixed connection resulted in higher stiffness of the overall GFRP truss bridge than the bolted span at both short and long serviceability [17,18]. It is widely known that the joints of a GFRP truss bridge restrict the development of the truss bridge [19,20,21,22], and so proposing GFRP joints with a high capacity and stiffness is of pivotal importance. 

In order to research the performance of GFRP joints, tests can be divided into two categories: (i) single- or double-lap direct shear tests of GFRP plates, and (ii) loading tests on full- or large-scale GFRP joints [23,24]. Direct shear test results showed that a bolted–bonded mixed connection has less deformation than bolted or bonded connections, but their ultimate capacity is very similar because they have the same failure modes [25,26]. Four typical failure modes were observed in the tests of bolted pultruded GFRP profiles: bolt bearing failure, GFRP plate tensile failure, GFRP plate shear failure, and GFRP plate splitting failure [27,28,29]. The load capacity and failure mode of the GFRP plate are related to the geometric dimensions such as hole diameter (φ), width of the GFRP plate (w), end distance of the GFRP plate (e) and fiber layup of the GFRP plate [30,31,32,33].

Compared with the direct shear test, the experimental research on full- or large-scale GFRP joints is scare. Chen carried out an experimental test and numerical analysis on a GFRP truss bridge where gusset plates were used in the joints [34]. The results showed that the gusset plate reduced the maximum stress occurring in the middle of the bottom surface of the lower chord of the midspan section. The stress value was far lower than the material strength, which can meet the needs of practical use. Ascione et al. [35] and Russo [36,37] proposed the use of pultruded profile gusset plates to connect more chords, but the end shear splitting damage of profile gusset plates was more severe under loading. In summary, the failure of a pultruded GFRP truss bridge mainly depends on the connection strength at the joint because of the local stress concentration around the connection region, but the research on full-scale pultruded GFRP profiles joint is also not extensive enough at present [38,39,40,41]. Whether the simple direct shear test can accurately reflect the real joints’ loading transferring paths in field applications of GFRP trusses and how it is related to the full- or large-scale GFRP joints’ test are still not clear currently [42,43,44]. Additionally, how the gusset plates change the load response of the GFRP joints is not clear at present [45,46].

In view of the above problems, a novel bolted integrated gusset plate connection is proposed and is expected to achieve better stiffness and capacity than typical a bolted connection and a bolted connection with a gusset plate. Considering the lack of research on bolted–bonded hybrid connections, this study presents an experimental and numerical investigation of a double-lap direct shear test. Full-scale testing of the proposed integrated gusset plate connection was conducted. The relationship between the double-lap direct shear test and the full-scale test of the connection is analyzed.

## 2. Materials and Methods

### 2.1. Materials

**Figure 1:**
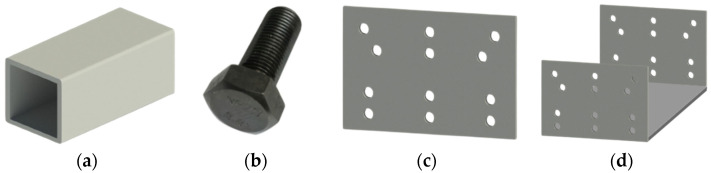
Materials used in tests of this study: (**a**) pultruded GFRP profiles, (**b**) steel bolt, (**c**) gusset plate, and (**d**) proposed integrated gusset plate.

**Figure 2:**
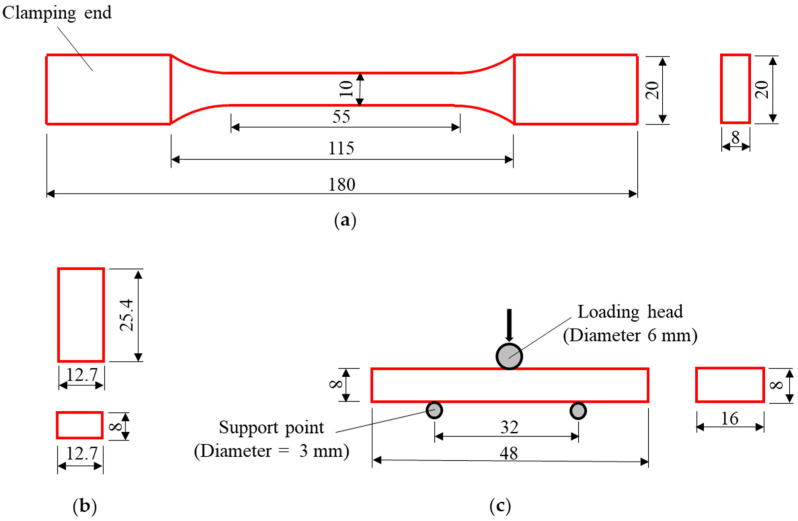
Geometric sizes of material test coupons: (**a**) tensile test, (**b**) compression test, and (**c**) shear test (units in mm).

The materials of the GFRP pultrusion profiles used in the tests (Figure 1a) are commercially available products from Nanjing Kangte composite materials company in Nanjing China. The resin used is unsaturated resin and the fiber is glass fiber. The material properties of the specimens were obtained directly from the walls of the pultruded profiles by an automatic CNC engraving machine (Tianxiang Inc., Tengzhou, China). Five tensile tests along and perpendicular to the fiber direction (Figure 2a), five compression tests (Figure 2b) and five longitudinal interlaminar shear tests (Figure 2c) were completed, and the results used were the average of the tests. The tensile strength of the GFRP profile was 308 MPa, the compressive strength was 210 MPa and the interlayer shear strength was 18.2 MPa, which were experimentally obtained according to Chinese code [47]. The modulus of elasticity of GFRP was 46 GPa according to the manufacturer. The bolts (see Figure 1b) used in the test were external hexagonal grade 8.8 high-strength M12 bolts, with a tensile strength of 800 MPa and yield strength of 640 MPa [12]. The modulus of elasticity of steel bolts was 210 GPa according to the manufacturer. The structural adhesive was an epoxy resin adhesive with shear strength ≥ 12 MPa and temperature resistance of −60 °C to 120 °C according to the manufacturer. 

The GFRP and high-strength bolts used in the three GFRP truss tests were the same as in the double-lap direct shear tests. The Q235 steel gusset plate (see Figure 1c,d) was used to combine different members in the truss series, with a density of 7850 kg/m^3^ and a modulus of elasticity of 200 GPa.

### 2.2. Methods

#### 2.2.1. Double-Lap Direct Shear Test of Bolted GFRP Plates

##### Specimen Design of Double-Lap Direct Shear Test

Referring to the experiments of Russo et al. [19,33], this study selected the number of bolts, plate width, plate end distance, plate thickness, and adhesive as variables and designed nine groups of GFRP profile bolted connection specimens. Test parameters and specimen configurations are shown in Table 1. Among them, the T1 specimen was a thick plate standard piece, and the changes in the T2–T9 specimens were as follows: T2—reduced the plate width *w*, T3—reduced the end distance *e*, T4—increased *e*, T5—added adhesive at the interface, T6—reduced the plate thickness *t*, T7—reduced t and used a single bolt, T8—reduced *t* and used three bolts, T9—reduced *t* and added adhesive at the interface. 

##### Construction of Double-Lap Direct Shear Test Specimens

**Figure 3:**
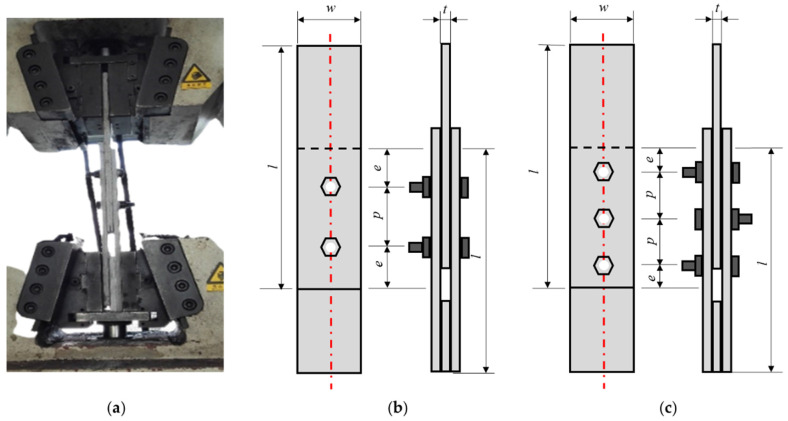
Double-lap shear test setup and geometric configuration of specimens: (**a**) actual photo, (**b**) specimen with two bolts, and (**c**) specimen with three bolts.

Double-lap direct shear specimens were obtained directly from the flanges and webs of the pultruded profiles by an automatic CNC engraving machine (Tianxiang Inc., Tengzhou, China). Then, the locations of bolts were marked, and the drilling was completed (as shown in Figure 3). The bolts were assembled by a trench that can measure the torque which is related to the pre-tightening stress within the bolts. For bolted mixed GFRP members, before tightening the bolts, the overlapping part was sanded and then evenly coated with epoxy resin adhesive to ensure sufficient contact between the adhesive and the surface.

##### Data Acquisition of Double-Lap Direct Shear Test

The GFRP double-lap direct shear connector test (Figure 3) used the SANS-SHT4106 universal testing machine (Sansi Eternal Technology Co., Ningbo, China). In order to eliminate the possible gap between the bolt and the plate, it was first loaded at a speed of 2 mm/min to reach 20 kN and then unloaded, and finally the main loading started at a loading speed of 2 mm/min. The test was terminated once the part was damaged or the specimen experienced a large displacement.

#### 2.2.2. Full-Scale Test of GFRP Pultruded Profile Truss Joints

##### Specimen Design of Full-Scale Test of GFRP Pultruded Profile Truss Joints

**Figure 4:**
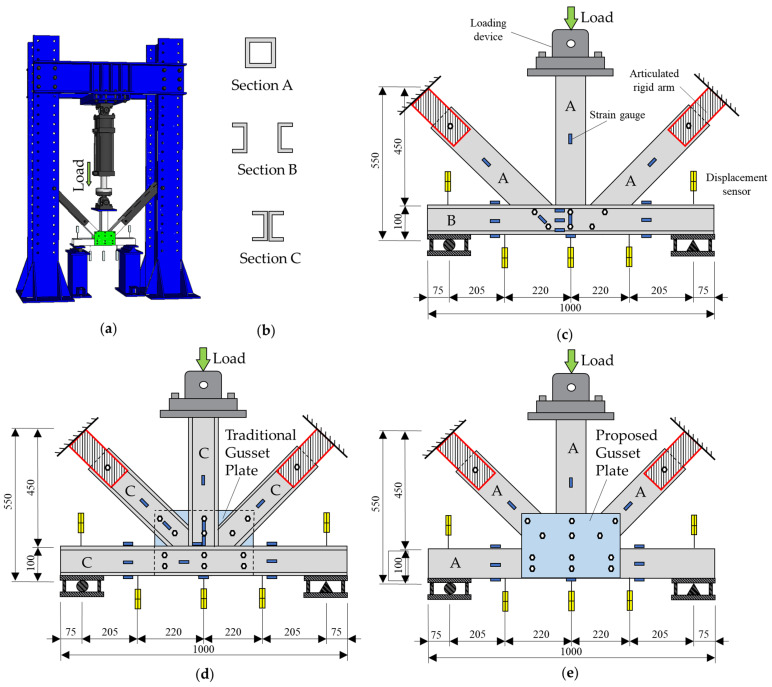
Full-scale test of GFRP truss joint: (**a**) test setup, (**b**) cross sections of members, (**c**) specimen BJ, (**d**) specimen GP, and (**e**) specimen IGP.

One full-scale typical clamping truss joint (specimen BJ, which stands for bolted joints), one typical gusset plate connection joint (specimen GP, which stands for bolted gusset plate joint), and one proposed integrated gusset plate connection joint (specimen IGP, which stands for integrated gusset plate joint) were designed. Each specimen was composed of a single 1000 mm lower chord web chord, two 450 mm 45° diagonal chords, a single 450 mm vertical chord, and a steel gusset plate connection. The cross-sectional dimensions of the pultruded section bars used for the joint specimens were the same, all of which were 100 mm × 100 mm closed rectangular sections. In order to maintain the consistency in the number of pultruded profiles, the required groove chords were cut by a CNC machine along the axis of rectangular section square tubes. Among them, the web of bottom chords of the typical clamping truss joint consisted of two 1000 mm web chords with a sectional size of 50 mm × 100 mm. The steel gusset plate connection-type truss joint was composed of two 1000 mm profile channel profiles, the lower chord web chord, and four 450 mm profile channel profiles to form a 45° diagonal chord, and two 450 mm profile channel profiles to form a vertical web member. The sectional size of all profile channel profiles was 50 mm × 100 mm. The test setup can be seen in Figure 4a. The three GFRP pultruded profile truss joints are shown in Figure 4c–e.

##### Data Acquisition of Full-Scale Test of GFRP Pultruded Profile Truss Joints

A hydraulic servo actuator with a maximum range of 300 kN acted directly on the top of the vertical bar for loading. The displacement and strain data of the joint static performance test were recorded by a TDS-7130 high-speed static strain data acquisition system produced by Tokyo Institute of Instrumentation, Japan. Due to the rotation of the spherical joint at the loading end of the hydraulic servo actuator, the load eccentricity occurred in the test process. Before loading, the sub-solid high-precision horizontal laser instrument (accuracy error 1 mm within 5 m) was used to accurately locate the vertical axis of the actuator and the vertical chord of the specimen to ensure that the actuator was consistent with the vertical axis of the vertical chord. YHD-50 displacement measurement range is 50 mm, and the accuracy error is controlled within 0.05%.

During the loading process of the monotonic static test, the data of displacement and strain were collected. The deformation and load of the specimen under cracking loads were observed and recorded. The test loading mechanism was as follows: (i) we preloaded 2 kN to the specimen to inspect the setup and data acquisition system and then unloaded after ensuring that all instruments work normally; (ii) in the load test stage, a 2 mm/min displacement control mode was adopted for loading, and the structural response was monitored and saved in real time; (iii) after the specimen reached the ultimate load and failed, the specimen was unloaded gradually until the end of the test.

## 3. Results and Analysis of Double-Lap Shear Test

### 3.1. Failure Mode and Load Response of Double-Lap Direct Shear Test

**Figure 5:**
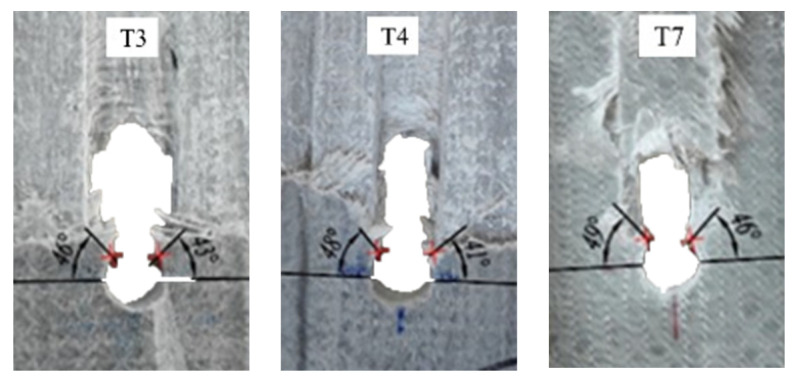
Shear failure at 45° of the bolt hole.

Three failure modes formed in the double-lap shear test, as shown in Table 2. It can be found that when the *w/φ* is small, the GFRP tensile specimen is prone to tensile failure. When the *w/φ* is large, the GFRP plate is prone to bearing failure along the adjacent region of bolt holes. Between the above two cases, shear failure occurred along the pultrusion direction. The observed results, without considering *e/φ*, are consistent with the situation of the test results of Okutan [33] and also prove the reliability of the test results and showed that most double-lap shear tests failed in bearing failure. The development direction of cracks grew about 45° along the bolt hole perimeter, as shown in Figure 5.

### 3.2. Parametric Study of Double-Lap Direct Shear Test

#### 3.2.1. Number of Bolts

With the increase in the number of bolts, the bearing capacity of the connector increased, but the effect of increased load-bearing capacity does not show a multiplicative relationship. Before the T6 specimen reached the peak load, the load continued to rise linearly and the stiffness was basically unchanged; at 15 kN (0.24$\;P_{u}$), due to the existence of bolt holes, the specimen experienced a small sudden drop in load, and the load returned to a linear rise quickly. When the peak load of the specimen was 62.1 kN ($P_{u}$), the load of the specimen decreased in a step shape. There were two descending plateaus, and the specimen showed good ductility characteristics.

In the early stage of the loading of the T7 specimen, the load basically increased linearly; at 8 kN (0.23$\;P_{u}$) and 13 kN (0.37$\;P_{u}$), due to the existence of bolt holes, the specimen had a small sudden drop in load, and the load returned to a linear rise quickly. When the load was continued, the specimen continued to make a fiber tearing sound. When the load was 31 kN (0.88$\;P_{u}$), the tensile stiffness of the specimen decreased. When the peak load of the specimen reached 35.2 kN, the load of the specimen suddenly dropped to 21 kN (0.60$\;P_{u}$), and there were three descending plateaus in total.

During the loading process of the T8 specimen, before 24 kN (0.37$\;P_{u}$), the load basically continued to rise linearly, and there was a sound of a fine fiber rupture during the loading process. Between 24 kN and 33 kN (0.50$\;P_{u}$), the stiffness of the specimen decreased due to the existence of bolt holes. After 33 kN, the stiffness of the specimen was restored. With the increasing of load, the specimen continued to make a fiber tearing sound until the peak load, and the specimen load maintained a linear increase. After reaching the peak load of 65.5 kN ($P_{u}$), the load of the specimen dropped suddenly and the displacement increased by 2 mm.

**Figure 6:**
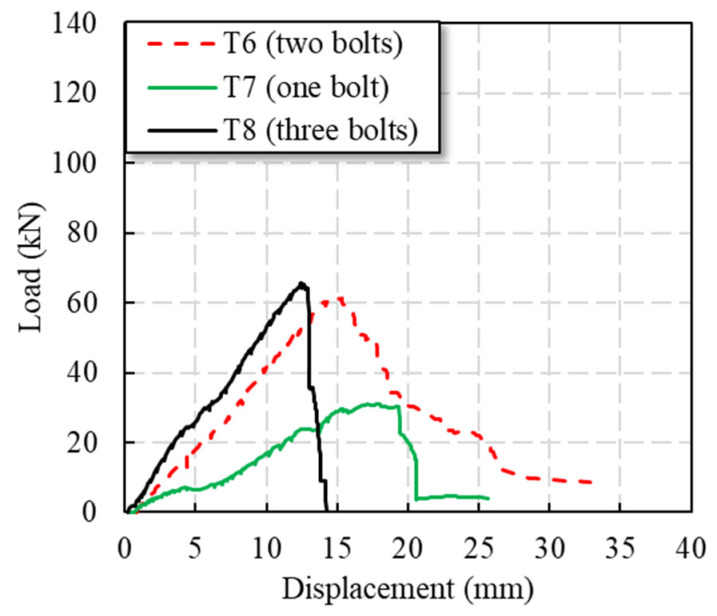
Load–displacement curves of specimens with different numbers of bolts.

It can be seen in Figure 6 that when the number of bolts increased from 1 to 2, the bearing capacity of a single bolt ($P_{u}$) increased significantly, and the double-lap direct shear thin plate connector increased by nearly 76.3%. At the same time, as shown in Table 2, T6 experiences tensile failure, while T7 experiences shear-out failure, indicating that shear-out failure can achieve a higher bearing capacity, which is the failure mode we prefer to obtain. When raising from a double bolt to three bolts, the double-lap direct shear bolted gusset plate connection $P_{u}$ only increased by 5.6%. It can be found from Figure 6 that the stiffness of the specimen was also increased with the increase in the number of bolts. Similarly, when the specimen was increased from a single bolt to a double bolt, the increase in the moving stiffness of the specimen was more obvious. The greater the number of bolts, the smaller the corresponding displacement when the specimen reached the ultimate load.

#### 3.2.2. Width of Plate

**Figure 7:**
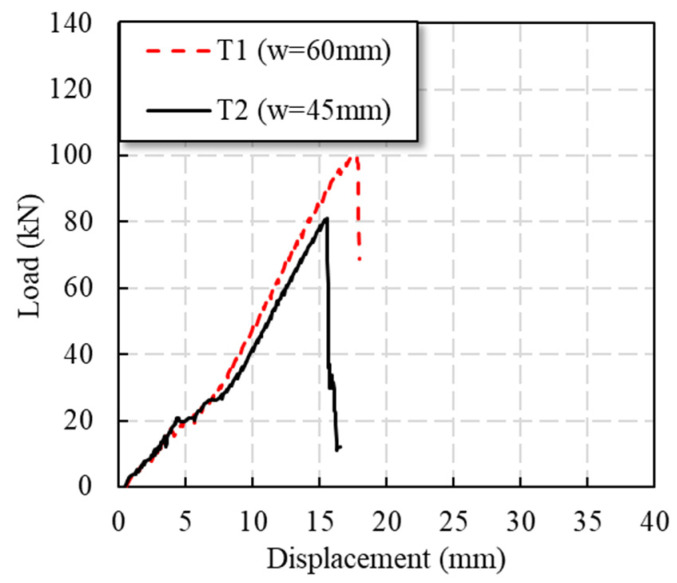
Load–displacement curves of specimens with different plate widths.

As Figure 7 shows, the T1 specimen basically maintained linear loading during the whole loading process. At 16 kN (0.16 $P_{u}$), there was a small sudden drop in load due to the existence of bolt holes. During the loading process after this, the specimen continuously emitted the sound of fibers tearing, which increased sharply in the later stage of loading. When the load reached the peak load of 100.7 kN ($P_{u}$), the specimen suddenly failed.

In the early stage of loading, the load of the T2 specimen basically continued to rise linearly. When it was loaded to 14 kN (0.17$\;P_{u}$), the specimen had a small sudden drop in load due to the existence of bolt holes. After 20 kN (0.25 $P_{u}$), the specimen had a load bearing plateau, and the displacement increased by 2.2 mm. After 30 kN (0.37$\;P_{u}$), the tensile stiffness of the specimen recovered and the load increased linearly. When the load was continued, the specimen continued to make a fiber tearing sound. When the peak load of the specimen was 80.8 kN ($P_{u}$), the specimen was suddenly damaged.

Table 1 shows that the ultimate bearing capacity of the specimens increased with the increase in *w/φ*, and the increase was more than 20%. With the increase in *w/φ*, the failure mode of the specimen changed: the GFRP plate of the T1 specimen has an obvious interlaminar shear effect when it is tensile, and its failure mode damage is that of bearing failure; the GFRP plate of the T2 specimen fractures along the bolt hole with interlaminar shear, and its failure mode is that of shear-out failure. It can be found from Figure 7 that the stiffness of the specimen with higher *w/φ* was improved to a certain extent in the later stage of loading. Compared with shear-out failure, the effect of bearing failure results in higher load capacity.

#### 3.2.3. Edge Distance of Plate

**Figure 8:**
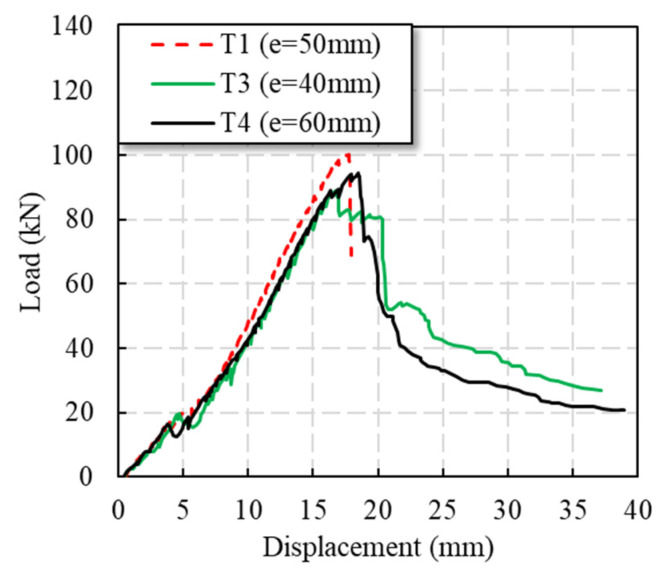
Load–displacement curves of specimens with different end distances.

The end distance is determined according to the size of the hole diameter. The control end distance/hole diameter (*e/φ*) ratio is 3.3 at minimum. The test phenomenon of the T3 and T4 specimens was similar to that of T1, but the T3 and T4 specimens had post-peak load responses until gradual failure, which was caused by the automatic stop of laboratory instruments in the T1 specimens. It can be seen from Table 1 and Figure 8 that the change in GFRP plate end distance between approximately 40 and 60 mm had little effect on load response, meaning that it can almost be ignored.

#### 3.2.4. Thickness of Plate

**Figure 9:**
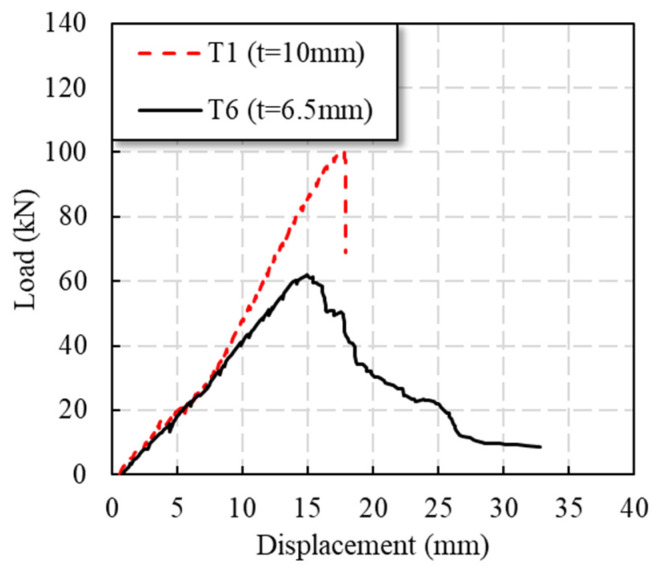
Load–displacement curves of specimens with different plate thicknesses.

It can be seen from Table 1 that the thickness of the GFRP plate can improve the $P_{u}$ of specimens, which can also be found in Figure 9. The failure mode of T6 is tensile failure. The peak load of specimen T1 was 62.3% higher than that of T6. Figure 9 shows that the stiffness of the T1 specimen was significantly increased compared with the T6 specimen after 40 kN. However, the T1 specimen did not show post-peak response, whereas the load of specimen T6 gradually decreased after the peak load. 

#### 3.2.5. Adhesive

**Figure 10:**
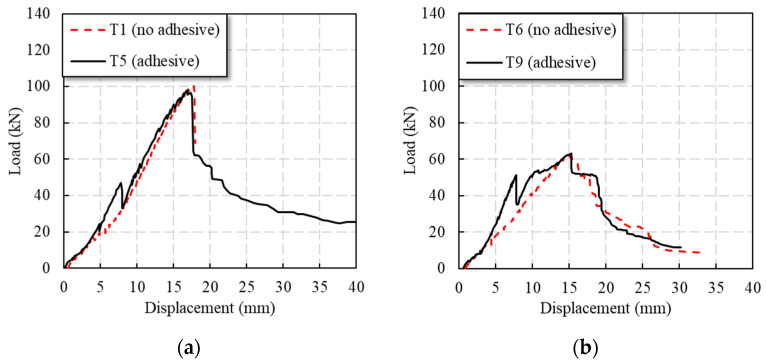
Load–displacement curves for comparison of the bolted and bolted–bonded hybrid connection: (**a**) thick plate, and (**b**) thin plate.

As Figure 10a shows, during the loading process of the T5 specimen, before 10 kN (0.10$\;P_{u}$), the load basically continued to rise linearly, and the initial tensile stiffness of the specimen was small; after 10 kN, the stiffness of the specimen increased slightly. When the load reached 49 kN (0.50$\;P_{u}$), the adhesive layer was shear damaged, and the specimen made a loud cracking sound. At the same time, the load suddenly dropped to 33 kN (0.34$\;P_{u}$), and then the stiffness of the specimen recovered, and the load continued to rise linearly. With continued loading, the specimen made a crisp sound of fiber tearing; when approaching the peak load of the specimen, the fiber emitted a continuous fiber tearing sound; after the peak load of 97.9 kN ($P_{u}$), the load suddenly dropped to 64 kN, and then the load decreased in a curve. In the later stage of load reduction, the specimen showed good ductility.

As Figure 10a shows, the stiffness of the T9 specimen was relatively small before 10 kN (0.16$\;P_{u}$). After 10 kN, the stiffness of the specimen increased significantly and the load increased linearly. When the load was 50 kN (0.79$\;P_{u}$), due to the shear failure of the adhesive layer, there was a loud cracking sound, and the load suddenly dropped to 35 kN. Then, the load continued to rise, but the stiffness of the specimen decreased. When the load was 54 kN (0.85$\;P_{u}$), the specimen slipped, and the tensile stiffness of the specimen was further reduced. When the load was continued, the specimen emitted a continuous fiber tearing sound. After reaching the peak load of 63.2 kN ($P_{u}$), the load decreased, where two descending plateaus were observed.

It can be seen from Table 1 and Figure 10 that the interface adhesive did not increase the specimen’s $P_{u}$. The initial stiffness of the specimen was significantly increased. When the load reached the shear strength of the adhesive layer, the load of the specimen decreased obviously. In the later stage of loading, the specimen mainly depended on the bolt to bear the load, so the $P_{u}$ of the specimen was basically the same as the pure bolted connection. The specimens T6 and T9 have similar shear strength, but it can be clearly observed that the adhesive layer failure occurred when T9 was about 50 kN. At the same time, comparing the glued specimens in the two groups of thick and thin plates (the same plate width and the same adhesive layer area), it can be found that the adhesive layer shear failure occurred in the specimens at about 50 kN, indicating that the shear bearing capacity of the adhesive layer in the same area was the same.

### 3.3. Numerical Simulation of Double-Lap Direct Shear Test

#### 3.3.1. Configuration of Finite Element Model of Double-Lap Direct Shear Test

**Figure 11:**
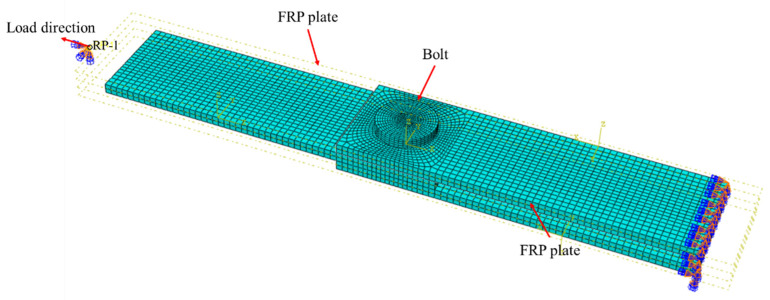
FE model of double-lap shear test of GFRP bolted joints.

A finite element (FE) model of the GFRP bolted joint was established in this study by using Abaqus, as shown in Figure 11. The bolt shank, nut, washer and GFRP plates were modeled according to the dimensions in Table 1 in the FE model, where a similar method has been shown in [48]. A reference point RP-1 was set on one end of the FE model of the GFRP bolted joint, and the reference point was coupled with the end surface, and the other end was constrained by consolidation.

The C3D8R element was used for the overall structure of the bolt, the density was 7850 kg/m^3^, the Young’s modulus was 200 GPa and the Poisson’s ratio was 0.1. The SC8R element was used for the GFRP plate, the density was 2700 kg/m^3^, the longitudinal Young’s modulus was 46 GPa, the transversal Young’s modulus was 9.0 GPa, and the in-plane shear modulus was 4.7 GPa, according to the manufacturer. In the established general contact, the friction coefficient of tangential behavior was selected as 0.1, and the normal behavior was selected as ‘hard’ contact and allowed to separate after contact. The mesh refinement was performed on the part where the bolt was in contact with the GFRP plate, increasing the accuracy of the mesh sizes to 1mm. In order to divide a suitable and easy-to-calculate mesh, the bolt was divided into a hexahedral structure as a whole. The GFRP plate was divided using the advanced algorithm in the hexahedral swept division and allowed the use of mesh mapping where appropriate.

The loading process was divided into two stages that were the same as the experimental test. First, we applied the preload and then applied monotonically increasing displacement at the loading end. The loading was solved by the dynamic explicit solver. The progressive failure of GFRP followed the Hashin damage initiation criteria [49]. Cohesive elements (zero-thickness elements) were employed to simulate the bonding layer in bolted–bonded specimens. In cohesive behavior, Knn was 30,000, Kss was 15,000, and Ktt was 15,000. In the damage attribute setting, the secondary tension criterion was selected. The damage of the cohesive element was output in the solution result.

#### 3.3.2. Comparison between FE Model and Experimental Results of Double-Lap Direct Shear Test

**Figure 12:**
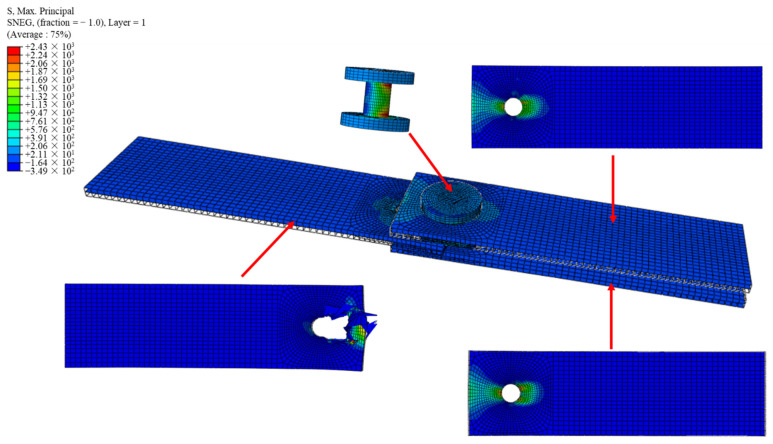
Maximum principal stress cloud of double−lap shear test of GFRP bolted joints.

**Figure 13:**
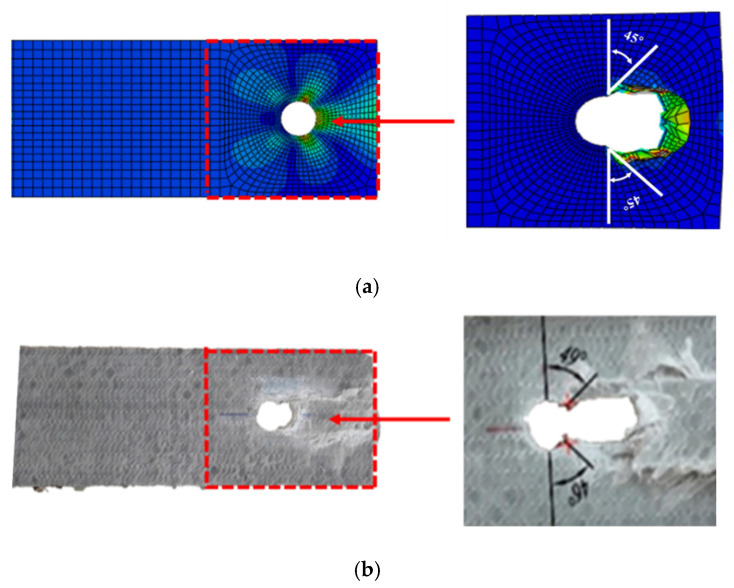
Comparison of (**a**) shear stress predicted by FE model, and (**b**) shear out failure mode of specimen T7.

The FE simulation results (Figure 12) show that the stress concentration phenomenon occurred in the 45° direction of the hole in the GFRP bolt connection. The failure mode matched well with the experimental results, as shown in Figure 13. In the experimental observations, the failure modes of T7 are both bearing failure and shear-out failure, which was also found in the results of numerical simulations, as shown in Figure 12 and Figure 13. The simulated failure process can be extracted frame by frame, providing a more complete picture of the damage process of the specimen than can be achieved in a test. It should be noted that a deformation magnification factor of 8.5 times was selected in Figure 12 and Figure 13.

**Figure 14:**
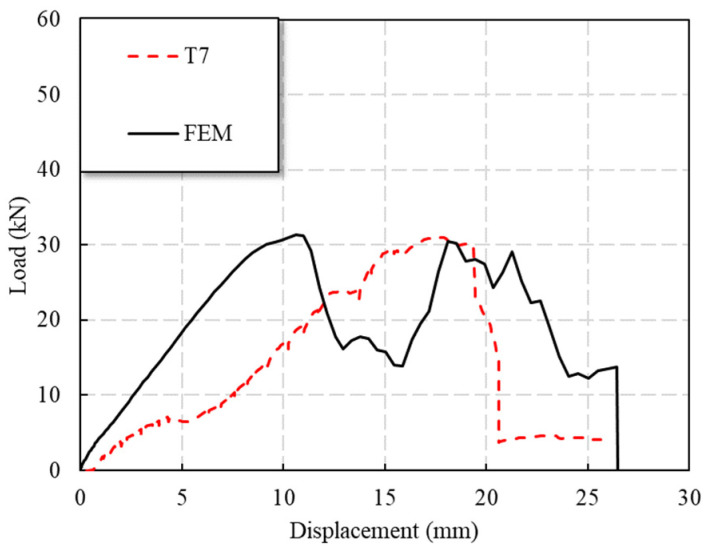
Comparison of FEM and test results of load response of specimen T7.

Figure 14 shows that the results of the numerical simulation were close to the results of the experimental test in terms of the maximum load. However, the displacement corresponding to the maximum test load was about 5 mm larger than the simulation results, which might be due to the initial gap between the loading head and the GFRP plate specimen.

#### 3.3.3. Analysis of Progressive Failure of Double-Lap Direct Shear Test Specimens

**Figure 15:**
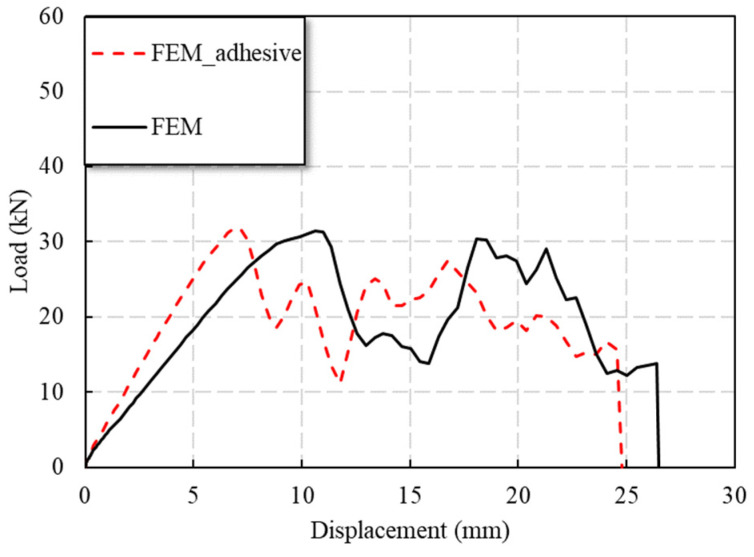
Comparison of FE model results: bolted and bolted–bonded hybrid connection.

Referring to the numerical model of the T7 specimen, the numerical model of the double shear single bolt with adhesives was established. As can be seen from Figure 15, the FE model of specimens with adhesives showed a higher initial stiffness of the specimen, and the load was significantly reduced after the failure of the adhesive layer. The load response predicted by the FE model was in good agreement with the experimental observations of the double-shear connection specimens T5 and T9.

**Figure 16:**
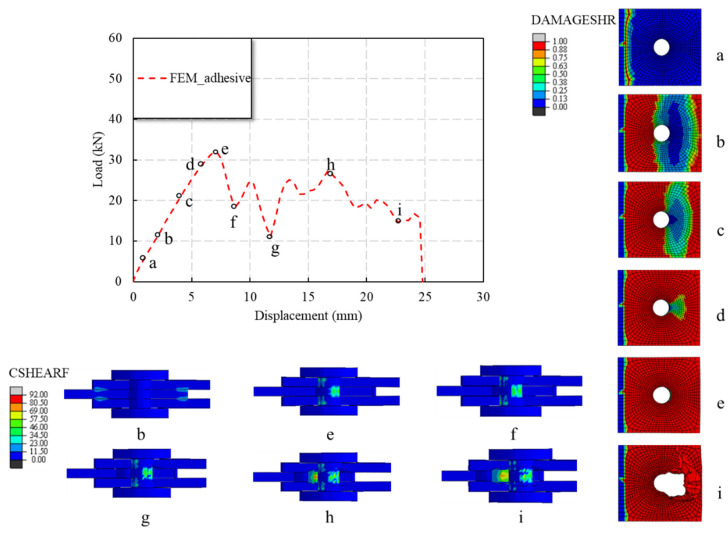
Progressive failure of the bolted–bonded hybrid connection specimen.

Figure 16 demonstrates that the adhesive on the loading end side first failed (see Point A in the load–displacement curve in Figure 16), and then the failure of adhesive near the fixed end was observed. As the displacement increased, the interfacial damage on one side of the fixed end extended to both sides and the middle. With the increase in displacement, the load reached a plateau after the peak of the load. This plateau, according to the authors’ analysis, is due to the progressive failure of the interfacial adhesive, as can be shown by Points F, G, and H in the load–displacement curve in Figure 16. This process was not apparent during the experiments, but some acoustic activities were heard during the test. Finally, the adhesive layer failed completely at the end of the post-peak plateau of the load (see Point I in the load–displacement curve in Figure 16).

## 4. Results and Analysis of Full-Scale GFRP Joint Test 

### 4.1. Tests Results and Observations of Full-Scale GFRP Joint Test

**Figure 17:**
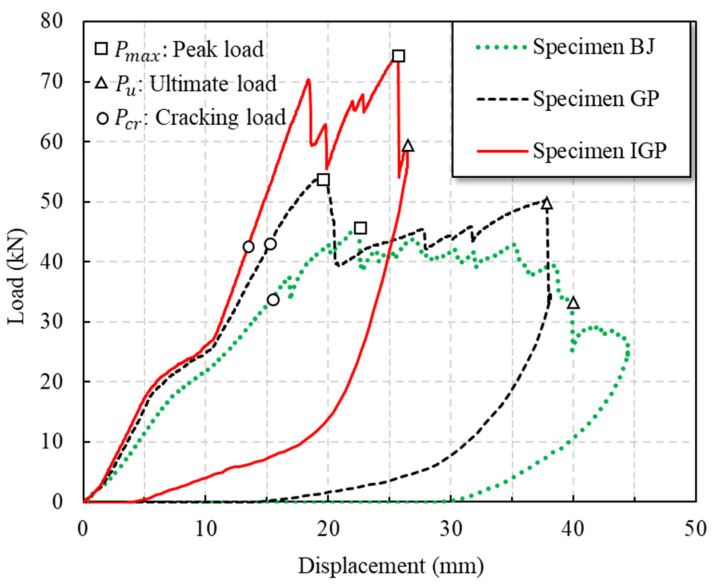
Load–displacement curve of GFRP truss joints.

The load–midspan displacement curves of specimen BJ, GP and IGP are shown in Figure 17. For specimen BJ, the first crack formed at the web–flange junction of the horizontal chord when the load reached 33.8 kN. At 37.4 kN, longitudinal shear cracks appeared between the upper row of bolts in the rear grooved web chord joint, while shear cracks formed at the hinge of the left diagonal chord. At 41.8 kN, multiple parallel longitudinal web shear cracks formed between the bolts of the front grooved web chord. When the bottom chord web chord reached the ultimate bearing capacity, the load dropped suddenly, and the end of the vertical chord extended out of the core area.

For specimen GP, cracks formed at the midspan flange of the front grooved web chord when the load reached 44.2 kN. With the increase in load, the top flange of the crack member collapsed and the deformation of the flange increased gradually. When the load reached 53.6 kN, the connection between the left web and the bottom plate of the front grooved web chord was fractured. After that, with the continuous increase in load, the vertical load was mainly carried by the hinged part of the diagonal chord. When the load decreased to around 48 kN, the pull-out failure occurred at the hinge of the right diagonal chord, resulting in the overall failure of the joint.

Specimen IGP experienced its first crack at the load of 42.33 kN at the web–flange junction of the horizontal chord. With the increase in load, the crack propagated along the vertical direction and finally resulted in overall failure at the ultimate load of 54.12 kN. The peak load of specimen IGP (74.47 kN) is 62% higher than the bolted one (specimen BJ), and 38.8% higher than the typical gusset plate joint (specimen GP).

Comparing the load–displacement curves of specimen BJ, GP and IGP (Figure 17), it can be seen that the typical bolted gusset plate (specimen GP) did not significantly increase the maximum load carrying capacity of the members. This might be caused by the fact that the lower chord pultruded profile was divided into two halves along the middle of the specimen in which the novel bolted gusset plate was incorporated. However, the inclusion of the novel bolted gusset plate significantly reduced the cracking and increased the ductility characteristics of the specimens by 21.62%. The employment of the proposed gusset plate substantially improved the peak load but reduced the ductility when compared with the rest two specimens. The reason for this improvement, according to the authors’ analysis, is that the proposed IGP works as a strengthening component of the joint and allows the bolts to work together to withstand the load. It is noted that ductility was estimated from the displacement to the point where the load dropped sharply after reaching the peak load.

The test results of specimen BJ, GP and IGP are shown in Table 3, where $P_{cr}$ and $\delta_{cr}$ are the load and displacement at the initial cracking of the vertical chord, respectively. $P_{d1}$ and $\delta_{d1}$ are the load and displacement when stiffness degradation occurred at the vertical chord, respectively. $P_{d2}$ and $\delta_{d2}$ are the load and displacement when stiffness degradation occurred, respectively. $P_{max}$ and $\delta_{max}$ are the peak load and corresponding displacement of the vertical chord. $P_{u}$ and $\delta_{u}$ are the ultimate load and corresponding displacement, respectively. Failure mode (a) represents the longitudinal shear of the web; (b) represents the shear and emergence of the end of the diagonal chord; (c) represents the failure of the bottom chord; (d) represents the buckling of gusset plate; (e) represents the bending of the bolt under compression; and (f) represents lower chord buckling.

### 4.2. Failure Modes of Full-Scale GFRP Joint Test

**Figure 18:**
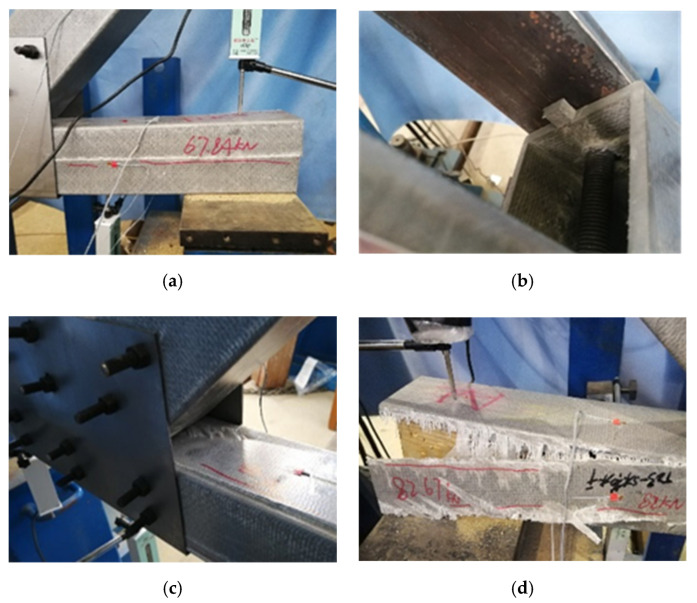

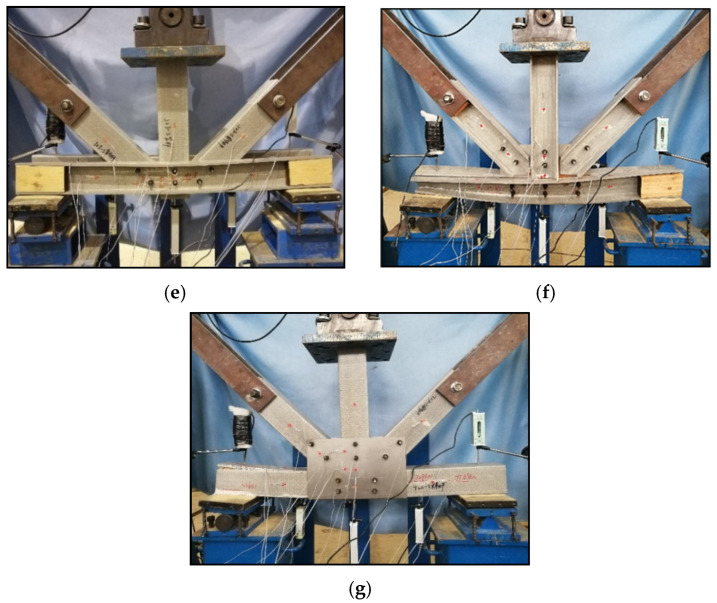
Failure modes of GFRP truss joints: (**a**) shear-out of webs, (**b**) shear-out of diagonal chords, (**c**) crush of top flanges, (**d**) separation of top flange and webs, (**e**) failed specimen BJ, (**f**) failed specimen GP, and (**g**) failed specimen IGP.

There were four typical failure modes in the static performance test of GFRP pultruded truss joints, as shown in Figure 18. Among them, specimen BJ showed shear out at the end of the diagonal chord, collapse of the top plate of the lower chord and buckling of the lower chord (see Figure 18e). Specimen GP was characterized by longitudinal shear of web, shear out of diagonal chord end and bottom chord buckling (see Figure 18f). Specimen IGP had a failure mode of shear failure at the web–flange junction (see Figure 18g).

**Figure 19:**
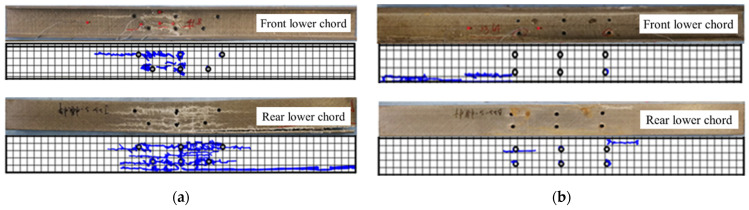

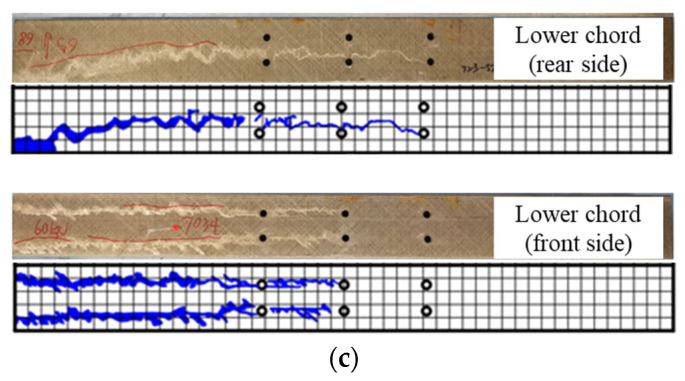
Distribution diagram of cracks in the lower chord: (**a**) specimen BJ, and (**b**) specimen GP, and (**c**) specimen IGP.

After disassembling the failure joints, the distribution of the static failure cracks of the bottom chord web is shown in Figure 19. A large number of longitudinal shear cracks appeared in the bottom web chord under the action of vertical load, and some large cracks extending into the core area of the joint directly led to the failure of the web chord. There was no full-length crack through the bottom chord. It should be noted that the distribution of all shear cracks was biased towards one side of the member. This was because the occurrence of shear cracks significantly reduced the stiffness of one side of the member, resulting in a small rotation of the joint around the core area, resulting in asymmetric failure on both sides of the lower chord web chord. The load orthogonal to the pultrusion direction of GFRP controls the mechanical resistance of the bolted connection, so it is recommended to add an adhesive between gusset plates and GFRP truss members to improve the capacity.

## 5. Comparison between Double-Lap Direct Shear Test and Full-Scale GFRP Joint Test

Both the double-lap direct shear test and the joint test can reflect the failure characteristics of GFRP pultruded bolted joints to a certain extent. Through comparison, it can be found that in the double-lap direct shear test and joint test, the failure of bolted joints mostly involves shear failure, and their cracks initiate at 45° around the hole and expand along the fiber pultrusion direction. However, there are more failure modes in the full-scale GFRP pultruded profile truss joint test, such as the collapse of the bottom chord roof and the failure of the web–flange connection, so it is necessary to carry out the full-scale test.

**Figure 20:**
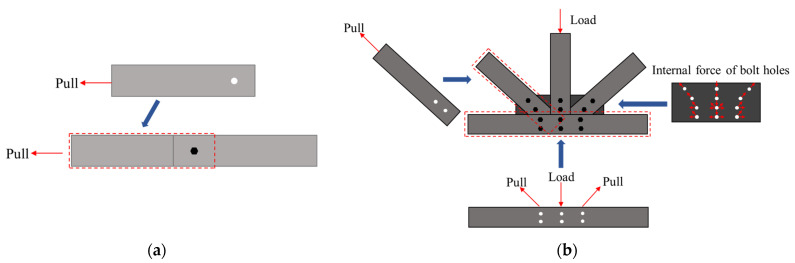
Failure modes of GFRP truss joints: (**a**) double-lap shear test, and (**b**) full-scale GFRP joint test.

In terms of the load transferring mechanism (Figure 20), the double-lap direct shear test specimen can represent the loading transferring from vertical and diagonal chords to the gusset plates and horizontal truss members. However, most failure modes occurred at the webs of the horizontal GFRP member, which was not well represented by the direct shear test. So, it can be concluded that the direct shear test can better replace the connecting capacity of vertical and diagonal chords. 

## 6. Conclusions

In this study, a novel integrated gusset plate connection was proposed for GFRP truss structures. The static tests of nine GFRP bolted connections and three full-scale GFRP pultruded profile truss joints were carried out. The effects of the number of bolts, the geometric parameters of the plate, and the interface adhesive on the bearing capacity and failure mode of GFRP bolted connections were studied. Through experiments and theoretical analysis, the following conclusions can be drawn:

1. The peak load of the specimen with the proposed integrated gusset plate is 62% higher than the bolted one, and 38.8% higher than the typical gusset plate joint. As a comparison, the typical gusset plate can delay the formation of initial cracks and substantially reduce cracks, but it does not significantly improve the bearing capacity of joints.
2. The bolted–bonded hybrid connection has 50% higher shear stiffness and 27% higher ductility compared with bolted joints, so the bolted–bonded hybrid connection is recommended to be employed together with the proposed integrated gusset plate.
3. Bearing failure is the primary failure mode for most specimens. A crack formed at the 45° direction around the hole and propagated along the pultrusion direction until failure. It is recommended that more fibers shall be added to the transversal direction of the FRP members to be connected.
4. Compared with bolted GFRP connections, the failure of GFRP pultruded profile truss joints is similar to the phenomenon in practical engineering of GFRP truss structures. The double-lap direct shear test specimen can represent the loading transferring from vertical and diagonal chords to the gusset plates and horizontal truss members.
5. This research showed the advantages of using integrated gusset plates of the joints of the GFRP truss. It should be noted that GFRP can be used in field applications to replace the steel gusset plate used in this experiment considering durability requirements.

## Figures and Tables

**Table 1:** Summary of the main parameters of GFRP bolted connection specimens.

Specimen ID	l (mm)	w (mm)	t (mm)	p (mm)	e (mm)	φ (mm)	$P_{u}$ (kN)	$P_{bolt}$ (kN)
T1	350	60	10	70	50	12	100.7	50.4
T2	350	45	10	70	50	12	80.8	40.4
T3	350	60	10	70	40	12	89.3	44.7
T4	350	60	10	70	60	12	94.5	47.3
T5	350	60	10	70	50	12	97.9	48.9
T6	350	60	6.5	70	50	12	62.1	31.0
T7	350	60	6.5	70	50	12	35.2	35.2
T8	420	60	6.5	70	50	12	65.5	21.8
T9	350	60	6.5	70	50	12	63.2	31.6

**Table 2:** Classification of failure modes of GFRP bolted joints.

Failure Mode	Specimen	Typical Damage Diagram
Bearing	T1, T3, T4, T5, T7	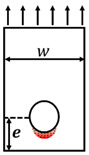
Tensile failure	T6, T9	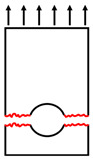
Shear-out	T2, T7, T8	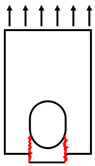

**Table 3:** Summary of static performance test results of GFRP pultruded truss joints.

Specimen	Failure Mode	$P_{cr}$ /kN	$\delta_{cr}$ /mm	$P_{d1}$ /kN	$\delta_{d1}$ /mm	$P_{d2}$ /kN	$\delta_{d2}$ /mm	$P_{max}$ /kN	$\delta_{max}$ /mm	$P_{u}$ /kN	$\delta_{u}$ /mm
BJ	(a) (b) (d)	33.75	15.36	17.43	7.25	22.53	10.48	45.78	22.44	36.33	38.73
GP	(b) (c) (d)	44.22	15.53	17.95	5.62	25.57	10.53	53.66	18.97	33.48	37.97
IGP	(a) (b) (c)	42.23	13.40	17.25	4.99	27.11	10.63	74.47	25.68	54.12	25.76

## Data Availability

Data sharing not applicable.

## Nomenclature

*e*	end distance of bolted pultruded GFRP specimen;
FRP	fiber-reinforced polymer;
GFRP	glass fiber-reinforced polymer;
IGP	integrated gusset plate;
*l*	length of pultruded GFRP specimen;
*p*	longitudinal spacing of bolts;
*t*	thickness of pultruded GFRP specimen;
*w*	width of pultruded GFRP specimen;
*φ*	hole diameter;
*P_bolt_*	ultimate load of a single bolt;
*P_u_*	load at the ultimate point;
*P_cr_*	load at the initial cracking point;
*P*_*d*1_	load at the stiffness degradation starting point;
*P*_*d*2_	load at the stiffness degradation end point;
*P_max_*	load at the peak point;
*δ_cr_*	displacement at the initial cracking point;
*δ*_*d*1_	displacement at the stiffness degradation starting point;
*δ*_*d*2_	displacement at the stiffness degradation end point;
*δ_max_*	displacement at the peak point;
*δ_u_*	displacement at the ultimate point.
